# Investigating the effect of synchronized movement on toddlers’ word learning

**DOI:** 10.3389/fpsyg.2022.1008404

**Published:** 2022-11-24

**Authors:** Marina Bazhydai, Han Ke, Hannah Thomas, Malcolm K. Y. Wong, Gert Westermann

**Affiliations:** ^1^Department of Psychology, Lancaster University, Lancaster, United Kingdom; ^2^Psychology School of Social Science, Nanyang Technological University, Singapore, Singapore

**Keywords:** toddlers, word learning, interpersonal synchrony, skin conductance, heart rate, behavioral synchronization

## Abstract

The effect of interpersonal behavioral synchrony on children’s behavior is an emerging field rich with research potential. While studies demonstrate its effect on affiliative and prosocial outcomes, the role of synchronized movement on children’s specific learning outcomes has not yet been investigated experimentally. One possibility is that synchrony, as a coordinated social activity, encourages perceived social bonds, leading to heightened attention, and better information retention. Equally likely is that physiological, rather than social learning, mechanisms mediate the effect, given the previously demonstrated role of autonomic arousal in attentional fluctuations, cognitive engagement, problem solving, exploration, and curiosity. The present study investigated the behavioral and physiological effects of synchrony conceptualized as induced, interpersonal, behavioral, movement-based interaction, on word learning in 2.5-year-old children. In a laboratory experiment, toddlers engaged in either a synchronous or an asynchronous movement-based interaction with an adult experimenter while listening to an upbeat children’s song. After the (a)synchronous movement episode, the same experimenter engaged children in a word learning task. During the (a)synchrony and learning phases, children’s physiological arousal was continuously recorded, resulting in heart rate and skin conductance response measures. Following a caregiver-child free play break, children were tested on their novel word retention. The results indicated that children learned novel labels at equal rates during the learning phase in both conditions, and their retention at test did not differ between conditions: although above chance retention of novel labels was found only following the synchronous, but not the asynchronous episode, the cross-episode comparisons did not reach statistical significance. Physiological arousal indices following the (a)synchrony episode did not differ between conditions and did not predict better word learning, although skin conductance response was higher during the learning than the movement episode. This study contributes to our understanding of the underlying cognitive and physiological mechanisms of interpersonal behavioral synchrony in the knowledge acquisition domain and paves the way to future investigations.

## Introduction

The focus of social cognition investigations has recently undergone a dramatic shift from isolated intra-personal responses to stimuli to inter-personal dynamic interactions (Davis et al., 2018; Hoehl et al., 2021). One main result of this research has been that synchronized activities lead to increased cooperative and prosocial behaviors. For instance, synchronized behavior with a stable pattern (i.e., engaging in joint actions such as drumming, finger tapping, clapping, jumping, or rocking) both in adults and young children is strongly associated with prosocial outcomes such as cooperation, helping, affiliation, bonding, interaction quality, rapport, likeability, and attachment (Hove and Risen, 2009; Miles et al., 2009; Valdesolo et al., 2010; Cirelli et al., 2014; Pearce et al., 2015; Rabinowitch and Knafo-Noam, 2015; Tunçgenç et al., 2015; Nguyen et al., 2020). However, this rapidly growing research field has not so far shed light on the role of interpersonal behavioral synchrony on cognitive rather than social outcomes. In the current study, we ask whether synchrony facilitates learning that occurs in social contexts but pertains specifically to the knowledge acquisition domain. We thus tested the effect of induced interpersonal synchronized movement on novel word learning in toddlers.

Studies with infants and children have reliably demonstrated the effects of synchrony on prosocial outcomes (Cirelli, 2018). One seminal study with 14-month-olds explored the effects of interpersonal movement synchrony on children’s prosocial behavior (Cirelli et al., 2014). To experimentally induce a state of (a)synchrony, infants were put in a front baby carrier and bounced either synchronously (in-phase and contingently) or asynchronously with an adult who stood in front of them while the infant listened to music. After the synchronous movement episode, infants were more likely to spontaneously help the experimenter in a prosocial task than after the asynchronous bouncing. In another study (Tunçgenç et al., 2015), 12-month-olds were rocked in a chair as they viewed a video of a toy (either a social one – a teddy bear that also established communication with the child, or a non-social one – a colorful box that produced sounds and lights) that was also positioned in a chair which rocked either synchronously or asynchronously with the child’s chair movement. When later given the opportunity to select one of them, infants preferred to reach to or crawl towards the toys that moved in synchrony with them only in the social, but not in the non-social condition. The prosocial effects of interpersonal movement synchrony also transfer to infants’ inferences and behavior towards adults uninvolved in the synchronous episode based on their social affiliations (Cirelli et al., 2016; Fawcett and Tunçgenç, 2017). Studies with preschoolers also showed that similarly induced synchrony enhanced children’s peer cooperation, imitation of, perceived similarity and closeness towards each other (Rabinowitch and Knafo-Noam, 2015; Rabinowitch and Meltzoff, 2017; O’Sullivan et al., 2018). In addition, the literature has documented the effect of synchrony on norm learning and ritualistic behavior (e.g., Herrmann et al., 2013), highlighting its importance for effective cumulative cultural knowledge transmission (Watson-Jones et al., 2021).

Extending and enriching these behavioral findings, a new generation of studies using hyperscanning approaches shows that behavioral synchrony leads to inter-brain synchrony through brain coupling (Dumas et al., 2010; Hasson et al., 2012; Hu et al., 2017). Early childhood studies using dual EEG and fNIRS approaches have reported that adults’ brains predictively synchronize to infants’ neural responses during social interaction (Leong et al., 2017; Wass et al., 2018) and support the conclusions that synchronized movements effect a range of interpersonal prosocial outcomes (Markova et al., 2019; Miller et al., 2019; Hoehl et al., 2021).

Unlike with prosocial outcomes, research into the effects of synchrony on specific learning outcomes so far is scarce and has produced inconclusive results. In a study with adults engaged in teaching and learning novel labels from each other following either a synchronous or asynchronous activity, synchrony did not lead to better word learning, although it had led to an increase in teacher-learner rapport (prosocial outcome) and inter-brain synchronization (Nozawa et al., 2019), in line with other hyperscanning studies with adults reporting associations between the brain activities of learners and teachers (Holper et al., 2013; Takeuchi et al., 2017). However, in another adult study, in addition to finding that teachers’ brains synchronized with learners’, the teaching outcome (here, numerical reasoning) was predicted by the interpersonal neural synchrony when the brain activity of the teacher preceded that of the learner (Zheng et al., 2018). Further, synchrony led to greater memory for details about people with whom participants were synchronized, but not greater generalized memory capacity (Miles et al., 2010). Overall, research with adults to date suggests a positive predictive role of the learner-teacher synchrony on learners’ engagement and attention during the explicit pedagogical process (Cheng et al., 2021), although this interim conclusion needs to be treated with caution due to mixed results and methodological inconsistencies (Hu et al., 2022).

Longitudinal studies with children showed that synchrony, broadly defined as responsive attunement, in infant-mother interaction predicted children’s subsequent school adjustment (Harrist et al., 1994) and verbal IQ (Feldman, 2007). Relatedly, though not measuring synchrony as such, specific learning outcomes such as vocabulary and math scores have been shown to benefit from teacher-child bonding (Lowenstein et al., 2015; Spilt et al., 2015; Roorda et al., 2017). Of crucial note, however, is substantial variability in the definition and conceptualization of synchrony in the developmental literature dealing with synchrony-related constructs (Harrist and Waugh, 2002). Broadly, one approach emphasizes contextual, cultural, and relational factors focusing on both inter-individual variability and intra-individual dynamics of behaviors (Jaffe et al., 2001; Feldman, 2006, 2012) and approaching synchronization as a complex dynamic system in development (Thelen and Smith, 1998; Mayo and Gordon, 2020). For example, when working together to solve a puzzle task, child-caregiver neural synchrony predicted coordinated problem-solving success in preschool children (Nguyen et al., 2020). On the other hand, there is also an extensive literature where synchrony in a dyad is conceptualized as temporal coordination, e.g., naming objects in synchrony with moving them in front of the child (Matatyaho and Gogate, 2008), with the effects of such turn-taking, intermodal, temporal synchrony on language development well documented (Rohlfing and Nomikou, 2014; Nomikou et al., 2016). For example, naturally occurring adult (both mother and stranger)-infant vocal rhythmic coupling at age 4 months predicted not only attachment, but also higher cognition scores on the Bayley Scales, at age 12 months (Jaffe et al., 2001). Nevertheless, while acknowledging these distinct and rich traditions in the study of synchrony, these only indirectly point to the hypothesized effect of interpersonal behavioral synchrony on learning in a specific knowledge acquisition sense. In sum, while interpersonal synchrony includes positive effects on children’s emotional and social experience (Leclère et al., 2014), its direct effects on specific memory and learning outcomes remain under-studied.

One of the greatest challenges facing researchers in this domain is identifying the underlying mechanisms behind the link between interpersonal synchrony and its outcomes (for reviews, see Cirelli, 2018; Davis et al., 2018; Hu et al., 2022). Broadly, two groups of mechanisms have been proposed: a higher-level socio-cognitive, and a lower-level neuro-physiological mechanism. The socio-cognitive, top-down process proposes that synchrony arises as a result of higher-level social perceptions or cognitive appraisals of the synchronous social situation (e.g., Baimel et al., 2015). Synchrony thus motivates prosocial behavior through forming domain-specific social representations. Another explanation, a bottom-up process, suggests that synchrony arises from neurobiological rhythms due to detecting perceptual contingency in mutual movement, gaze, and action such as finger tapping, or engaging in joint musical activity. In turn, this entrainment to social rhythms activates domain-general attention mechanisms in the social context and stimulates prosocial interactions (e.g., Markova et al., 2019). The two mechanisms may be complementary rather than alternative to each other, explaining the same phenomenon at different levels. Indeed, the evidence for these mechanisms is so far mixed and suggests that both processes might be at play at the same time. Moreover, as the investigated outcomes primarily related to prosociality and cooperation, the hypothesized mechanisms might be limited to these accumulated empirical findings.

Along with the effect of synchrony on prosocial outcomes, as part of the same mechanism there may be also an effect on specific learning outcomes in a social context. It is possible that synchrony also leads to heightened learning readiness or better encoding of information that was acquired while in the state of synchrony with the social partner. Within the top-down socio-cognitive framework this would be expected if higher-level affiliative judgements and perceived similarity due to enhanced and enriched social interaction during synchrony transfer to the learning domain. This stronger affiliation to the learning partner could then affect, for example, how learners evaluate information provided by others or their desire to live up to the expectations arising in direct pedagogical context, and lead to a higher chance of encoding new information. At the same time, bottom-up, biological synchronization may drive attention mechanisms, in that teachers who are in sync with learners may provide them with necessary attention modulation to keep them focused on learning, and such attention, as a lower-level attribute, may lead to better learning, and information retention.

### Study motivation

As detailed above, the effect of interpersonal synchrony on children’s behavior is a rapidly expanding field rich with research potential. Studies have shown that experiencing interpersonal synchrony encourages affiliative and prosocial behavior in children. However, the role of directly experienced behavioral synchrony on specific learning outcomes in early childhood has not yet been directly investigated experimentally. If such a relationship exists, there may be several possible cognitive mechanisms underlying it. One possibility is that synchrony, as a coordinated social activity, encourages perceived social bonds between the child and the adult, which leads to heightened attention and better information retention. Equally likely, the physiological, rather than social learning, mechanisms could be responsible for the hypothesized relationship. The proposed study aims to investigate if the effects of synchrony extend to learning, and if there are psychophysiological markers of it. Importantly, it was not designed to tease apart which of the two mechanisms is at play, but rather capture the effect of synchrony at both behavioral and physiological levels. Whichever causal mechanism is in place, it is plausible to expect that the increased physiological arousal associated with synchrony leads to higher rates of learning.

### The current study

For the purposes of this study, we conceptualized synchrony as a rhythmic movement to music occurring without the child’s active intention, but randomly assigned and controlled by others, which leads to achieving interpersonal synchrony with a stranger in a momentary interaction. This allowed us to isolate the process of synchronization in an experimental, highly controlled setting, and was in line with prior seminal experimental lab-based research with infants and young children, where synchrony was induced by a rocking chair, swing-set apparatus, or another person’s movement (Cirelli et al., 2014; Tunçgenç et al., 2015; Rabinowitch and Meltzoff, 2017).

We induced the experience of interpersonal (a)synchrony between 2.5-year-old children and the experimenter in a laboratory setting. Children sat on their caregiver’s lap. Both caregiver and experimenter wore headphones through which a song was played, either synchronized or not. The experimenter moved, and the caregiver rocked the child from side to side, according to the beat of the song they were listening to, resulting in either a synchronous or asynchronous movement between the child-caregiver dyad and the experimenter. Following this phase, children engaged in a novel word learning task facilitated by the same experimenter (Horst and Samuelson, 2008). During the synchrony and the learning episodes, we also measured children’s physiological arousal (measured by heart rate and skin conductance response signals derived from a wearable wristband device, Empatica E4) conceptualized as an index of heightened attention and interest.

We predicted that interpersonal behavioral synchrony would differentially affect children’s learning, in that following a synchrony episode, children would successfully retain more new words than following an asynchrony episode (Hypothesis 1). We further expected that physiological arousal level would be higher during synchrony (Hypothesis 2) and that at an individual level, heightened arousal would predict higher rates of successful word retention (Hypothesis 3).

## Materials and methods

### Participants

Participants were typically developing 2.5-3-year-old children (*N =* 40; 17 males, 23 females; *M*_age_ = 32.08 months, *SD*_age_ = 1.53 months; range 28–34 months) and their primary caregivers, recruited from a database of families in the Northwest of England who had voluntarily expressed interest in participating in infant studies. Participating families were reimbursed for travel expenses and children received a book as a gift, in accordance with standard laboratory practices. The study received university ethics committee approval and caregivers provided informed written consent. The experimental protocol was preregistered on April 15, 2019, prior to the start of the data collection and is available at^1^. Data collection took place between April 2019 and August 2019.

The sample size was determined *a priori* using the G*Power analysis software (Faul et al., 2007), which indicated that a sample size of *N* = 18 would be sufficient to produce a large effect size (with a power of 0.80 and alpha of 0.05) based on the main statistical analysis. We collected data from 40 children randomly assigned to two conditions (synchronous and asynchronous) with 20 children in each. Additional 7 participants were tested but their data excluded for the following reasons: child fussiness or refusal to take part in the procedure at any phase of the experimental design resulting in an incomplete dataset (*n* = 6) and technical error (missing video recording, *n* = 1).

### Experimental procedure and materials

Upon arriving to the laboratory, caregivers received instructions during the consenting procedure. The child, upon their verbal assent, was fitted with an Empatica E4 wearable wristband device to measure physiological arousal (heart rate and electrodermal activity)^2^ (Empatica Inc., 2015; a wearable research device validated in adults; van Lier et al., 2020) and successfully used in developmental and atypical populations (Mehr et al., 2017; Bainbridge et al., 2021). The band was positioned on the child’s leg as close to the foot as possible on either calf or ancle, depending on the size of the child, which was in line with other studies using the same research device and the manufacturer’s recommendations (Bainbridge et al., 2021). As an incentive to attaching the band, an attractive sticker of the child’s choice was placed on the band and the child was told that when they were all done, they would be allowed to keep the sticker.

**Figure 1:**
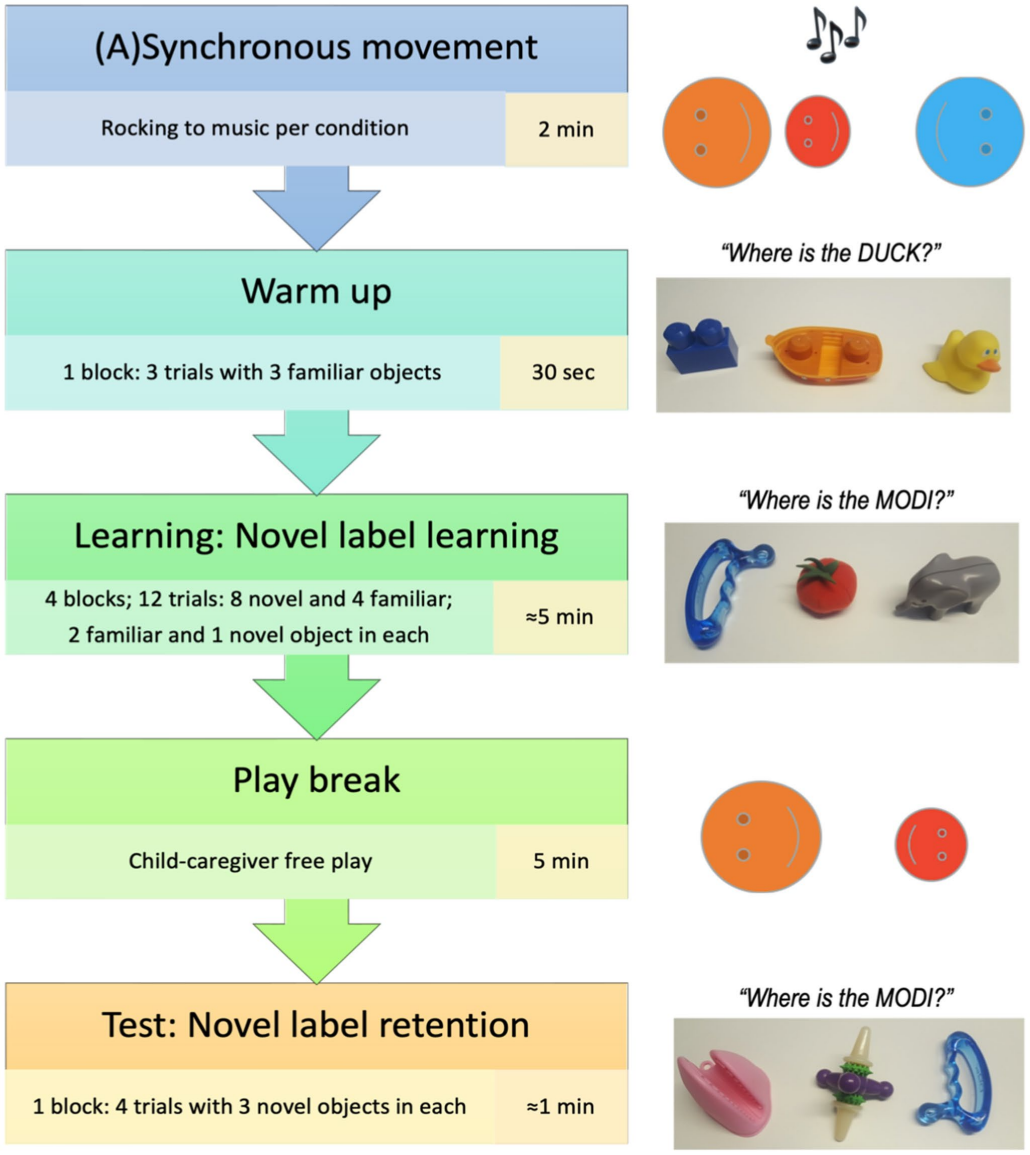
Experimental flow.

The experimental flow is presented in Figure 1. The procedure consisted of an interpersonal movement episode, followed by a warm-up and novel label learning phase, a play break, and concluded with the test phase to assess novel label retention. Children were randomly assigned to either a synchronous or an asynchronous condition using a between-subject design. The experiment was recorded using a single video camera and lasted approximately 15 min.

**Figure 2:**
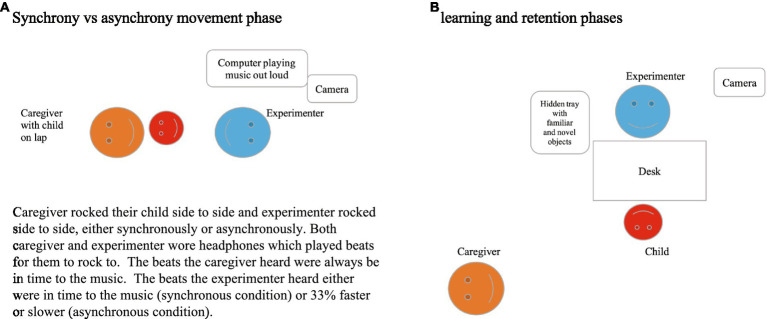
Experimental setup.

The interpersonal movement synchrony between the experimenter and the children was manipulated by asking the caregiver to playfully rock their child to a 2 min children’s song (the “Happy Song” by Imogen Heap^3^) played out loud with a constant beat of 84 bpm. Children sat on their caregiver’s lap on the floor, facing the experimenter who sat across from them on the floor at a distance of approximately 1 m (Figure 2).

Half of the sample (*n* = 20) experienced a synchronous movement episode with the experimenter. Specifically, their caregiver rocked the child side to side to the beat of the song (as heard through the programmed headphones) and the experimenter mirrored this, rocking side to side, to produce a synchronous movement episode. The other half of the sample (*n* = 20) was also rocked by their caregiver side to side to the beat of the song, however, the experimenter in this case rocked asynchronously – with beats either 33% faster or slower than the caregiver rocking the child to the song’s beat (adapted from Cirelli et al., 2014 design). In both conditions, both the parent and the experimenter wore headphones that played metronome beats to which they rocked side to side, but not the music. Children instead heard the song played out loud through the speakers, but not the metronome beats. The song’s rhythm and the caregiver’s rocking were always congruent to each other, while the experimenter’s rocking varied according to condition. The caregivers sat cross-legged with their child on their lap and created the sideways rocking motion by lifting either leg up slightly in alternating order. Caregivers were instructed to refrain from otherwise actively interacting with their child throughout the study, aside from completing the rocking motion and encouraging their child to engage with the experimenter if the child lost attention at any point. This episode lasted 2 min.

Following the (a)synchrony episode, children engaged in the word learning task with the same experimenter. They independently sat at a table across from the experimenter with the caregiver sitting behind and to the side of them (Figure 2), and the experimenter presented the tray with objects and labels.

The word learning task consisted of two phases: learning and retention test. The learning phase was a referent selection task (based on Horst and Samuelson, 2008), in which the child had an opportunity to learn 4 pseudo words (*koba, modi, blicket, and toma*) for 4 novel objects in trials where at each trial, two familiar objects were paired with one novel object and the novel label was introduced. Objects were presented by the experimenter on a tray with three sections next to each other. The warm-up block consisting of three trials familiarized the child to the referent selection procedure with three familiar objects, proceeding to the word learning task. On each learning trial the experimenter presented the tray with three objects, two familiar and one novel, and asked the child to choose a novel object labeled with the pseudo word (“I see a [familiar/novel object]! Can you see a [familiar/novel object]? Can you pass me the [familiar/novel object]?”). The experimenter made eye contact with the child upon presenting the tray and maintained the gaze on the child, not the tray, until the child made a selection (by pointing at it, touching it, reaching for it, or handing it over), and provided positive reinforcement to the child regardless of the selection. In total, children received four familiar and eight novel referent selection trials (2 for each novel object). The experimenter asked for the novel objects during the first two trials, and for the familiar objects during the last trial of each block, and repeated this process for each of the three novel object-label pairs in a pseudo-randomized order.

**Figure 3:**
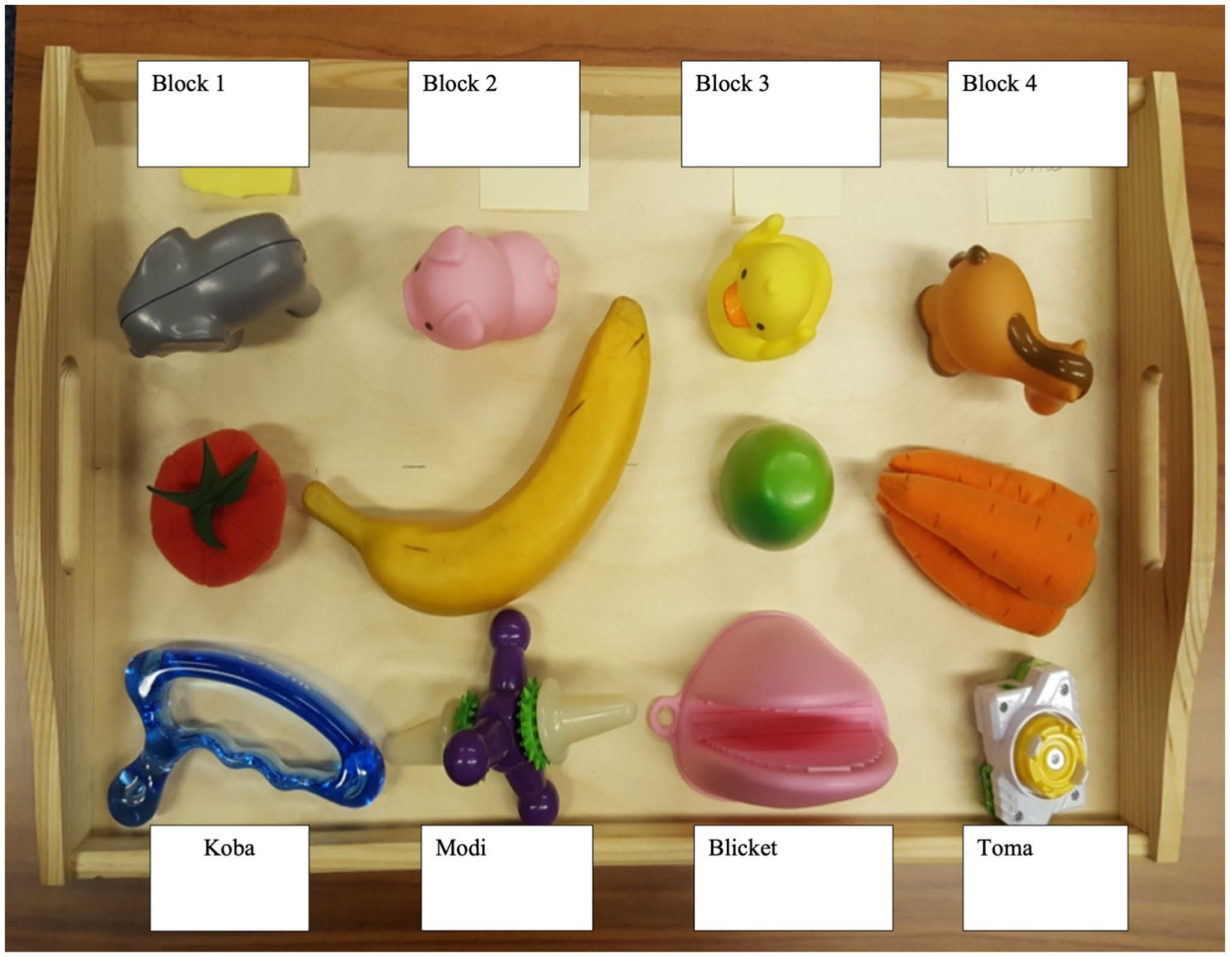
Learning phase: Novel label learning task objects.

Familiar objects selected for the task (Figure 3) were in line with the CDI norms data (Frank et al., 2016). The objects were selected from two categories – food items and animals – and grouped so that one object from each category was presented with the novel object during each trial of the referent selection phase.

Following this referent selection phase and prior to the retention phase was a free-play 5 minute episode during which the experimenter left the room, and the child could play with a range of toys on the floor of the testing room. Caregivers were explicitly asked to ensure that children did not approach the objects’ tray.

For the retention phase the experimenter returned to the room and the child again sat at the table across from the experimenter as during the learning phase. In the test phase, children’s retention of the learned referents was assessed. On each of the four test trials, the experimenter presented the tray containing three of the previously seen four novel objects, in a pseudo-randomized order, and asked for one of them (“I see a [pseudo word]! Can you see a [pseudo word]? Can you pass me the [pseudo word]?”) such that each novel object was asked for once. After choosing an object (by pointing at it, touching it, reaching for it, or handing it over) the child was thanked; no feedback on the correctness of the choice was given.

The physiological response was measured using the wristband continuously throughout the (a)synchronous episode and the word learning task, with the first event marker signifying the start of the (a)synchronous episode and the second event marker signifying the start of the word learning task. The experimenter removed the band before leaving the room for the free play break preceding the word recall phase.

## Measures and coding

### Manipulation check and *post hoc* behavioral control coding

To ensure the synchronous and asynchronous conditions were reliably achieved, we coded the degree of synchrony during the movement episode. First, two blind coders (with second coder coding 20% of the participants, *n* = 8) made a judgment of the condition based on the observed synchrony in the movement of the caregiver with the child and the experimenter. Second, the coders rated the level of synchronization in the dyads during the movement episode using a Likert-type scale (1 – absolutely non-synchronous, 4 – sometimes synchronous, sometimes non-synchronous, and 7 – absolutely synchronous). The raters’ agreement was very high, indicating that blind coders could reliably guess the condition (Kappa = 1 for condition guess and Cronbach’s alpha = 0.991 on ratings of synchronization). Confirming the successful manipulation check, the level of synchronization was significantly higher in the synchronous (*M* = 6.6, *SD* = 0.6) as compared to the asynchronous condition (*M* = 1.68, *SD* = 0.75), as demonstrated by an independent-samples *t*-test (38) = 22.7, *p* < 0.001].

Further, to check whether the experimenter displayed equal levels of positive affect (operationalized as the rate of smiling while making eye contact with the child) during the movement phase in both conditions, the coders assessed it using a Likert-type scale (frequency of smiling: 1 – very rarely; 2 – rarely; 3 – sometimes, 4 – often, and 5 – very often). The child’s positive affect was also coded in the same manner. The inter-rater reliability was very high (Cronbach’s alpha = 0.991 on both ratings). The results of the independent-samples *t*-test support the assumption that the experimenter’s positive affect was consistent between conditions, *t* (37) = 1.54, *p* = 0.066 (*M_sync_* = 5.0, *SD* = 0.0; *M_async_* = 4.89, *SD* = 0.32), and the children also displayed similar levels of smiling during both conditions, *t* (32) = −0.837, *p* = 0.204 (*M_sync_* = 2.06, *SD* = 1.4; *M_async_* = 2.5, *SD* = 1.63), confirming that the movement (a)synchronization was indeed the distinguishing feature of the condition assignment.

Finally, the free-play episode was also coded *post hoc* to ensure there was no mention of the preceding learning phase by the caregivers or children, which may have influenced the retention of the word-object pairs as measured at test. The coders noted the number of times the parent mentioned novel objects or words learned in Learning phase. The coders’ agreement was 100% and no caregiver in this sample mentioned the stimuli during this break.

### Behavioral measures

During the word learning task, children received four familiar and eight novel referent selection trials and four retention trials. The following three variables were computed: (1) The number of familiar objects correctly selected during the referent selection phase; (2) The number of novel target objects successfully selected during the referent selection phase; (3) The number of novel words successfully retained at test. The referent selection and object choices were coded offline from the video recordings, indicating whether the child selected the correct object (e.g., a novel object referred to as *koba* on the tray that presented it along with two familiar objects; see Figure 1) by pointing at it, touching it, reaching for it, or handing it over to the experimenter. The main coder coded 100% of the videos and the second coder coded 20% of the videos (*n* = 8; 4 from each condition), reaching perfect reliability (Cohen’s Kappa = 1). Both coders were blind to the analyzed condition (synchronous or asynchronous). For each of these outcome variables, the proportion of correct choices was calculated by dividing the number of correct responses by the total possible (i.e., 4 for familiar trials, 8 for novel trials, and 4 for retention trials).

### Physiological measures

The physiological response data acquired during both phases (synchrony and word learning) were averaged to produce the heart rate and skin conductance level indices during the (a)synchrony and the word learning phases. Each sensor’s sample rate was embedded in the output provided by the manufacturer and optimized to capture the frequency content of relevant signals (Empatica Inc., 2015).^4^

The heart rate data from Empatica E4 is indexed as blood volume pulse (BVP; sampled at 64 Hz) with data from photoplethysmograph (PPG) and inter beat intervals (IBI; intermittent output with 1/64 s resolution). We used inter-beat intervals as our primary measure; these were computed by Empatica’s proprietary algorithm, which automatically imputes missing data from the photoplethysmograph signal and corrects for motion artifacts; with some segments in time devoid of IBI data (refer to Empatica E4 wristband User’s Manual). We first identified the data points that corresponded in time to each of the two experimental phases (movement episode and word learning phase), as signified by two event markers recorded through the manual presses on the physiology band by the experimenter. To make the IBI data correspond to the EDA data on a time scale, we extrapolated the IBI data to 4 samples per second by computing duplicate values if needed. We selected a baseline period of 30 s immediately before the start of the movement phase. We then averaged z-scored values for each experimental phase and computed a difference score between averaged values and baseline values to control for individual differences.

The data from the electrodermal activity sensor (EDA, sampled at 4 Hz in μS, i.e., four samples per second) was indexed as the basal tonic skin conductance level (SCL), which is relatively stable and associated with gradual changes in skin conductance. We subtracted the skin conductance response amplitudes from the tonic signal to establish a better representation of SCL. The Ledalab^5^ software based on MATLAB (The MathWorks, Inc., Natick, MA, and United States) was used for raw signal processing as recommended by the wristband manufacturer. Signal processing submits raw data for decomposition analysis and feature extraction of the EDA signal. Extracted SCL data were visually inspected for movement artifacts (atypically large spikes or drops in the amplitude) and low signal quality which were excluded from the cumulative measures. We exported z-scored values from Ledalab, averaged z-scored values for each child’s SCL for each of the two experimental phases and computed the baseline-corrected difference score.

## Results

*Hypothesis 1.* Effect of synchrony on word learning.

**Figure 4:**
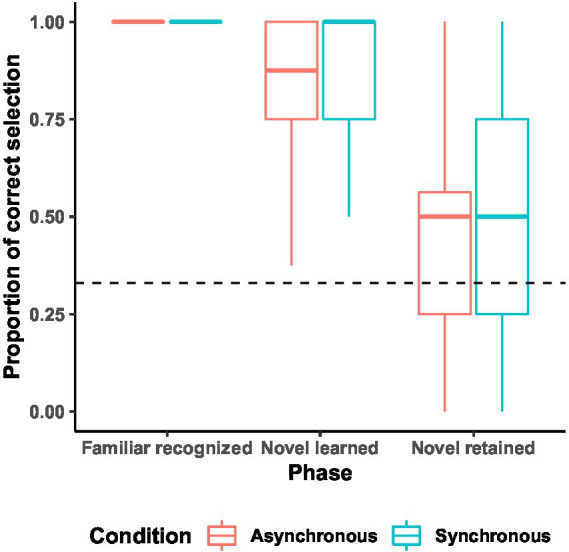
Proportion of retained words at test across word learning task phases. Boxplot of the proportion of the correctly selected objects in each of the three phases of the word learning task, with the dotted line indicating the chance level (0.33) and the solid lines indicating the median values.

All children correctly identified the familiar object-referent pairs. The proportions of correctly selected novel objects during the word learning task were as follows: novel objects selection at learning: Synchrony condition: *M* = 0.89, *SD* = 0.16; Asynchrony condition: *M* = 0.82, *SD* = 0.2; novel objects retention at test: Synchrony condition: *M* = 0.49, *SD* = 0.31; Asynchrony condition: *M* = 0.44, *SD* = 0.31 (Figure 4).

We pre-registered to conduct a 2 (condition: synchronous or asynchronous) × 3 (trial type: familiar recognized vs. novel learned vs. novel retained) mixed analysis of variance. As the assumptions for the parametric analysis were not met, we instead conducted the non-parametric equivalents and performed the Mann–Whitney tests on novel learned and novel retained phases. There were no significant differences between conditions in either phase; novel learned phase: Synchrony (*Mdn* = 1), Asynchrony (*Mdn* = 0.88), *U = 153, p* = 0.17; novel retained phase: both *Mdn* = 0.5, *U = 184.5, p* = 0.68, indicating that children learned and retained novel labels at equal rates in both synchronous and asynchronous movement conditions.

Next, we conducted one-sample *t*-tests to calculate if children retained the novel referents at proportions above chance, with the chance level set at 0.33 for all reported tests. Further, a Bayes Factor analysis was performed to obtain support for either the alternative or the null hypothesis for each of the main analyses with a half normal distribution (implying a maximum possible effect size of 0.707). For the Bayes Factor analyses, we used the system proposed by Jeffreys, (1961) to interpret the size of a BF: BF_01_ < 3 is considered moderate support for the null hypothesis, BF_10_ > 3 is considered moderate support for the alternative hypothesis. These analyses revealed the above-chance retention of novel labels only in the synchronous [*t*(19) = 2.28, *p* = 0.03; *BF_10_* = 2.65, *M* = 0.49, *SD* = 0.31], but not the asynchronous condition [*t* (19) = 1.54, *p* = 0.14; *BF_01_* = 1.14, *M* = 0.44, *SD* = 0.31]. Nevertheless, the Bayes Factors indicated insufficient support for either hypothesis.

We therefore conclude that we could not reject the null for our hypothesis 1.

*Hypothesis 2.* Effect of synchrony on physiological arousal.

**Figure 5:**
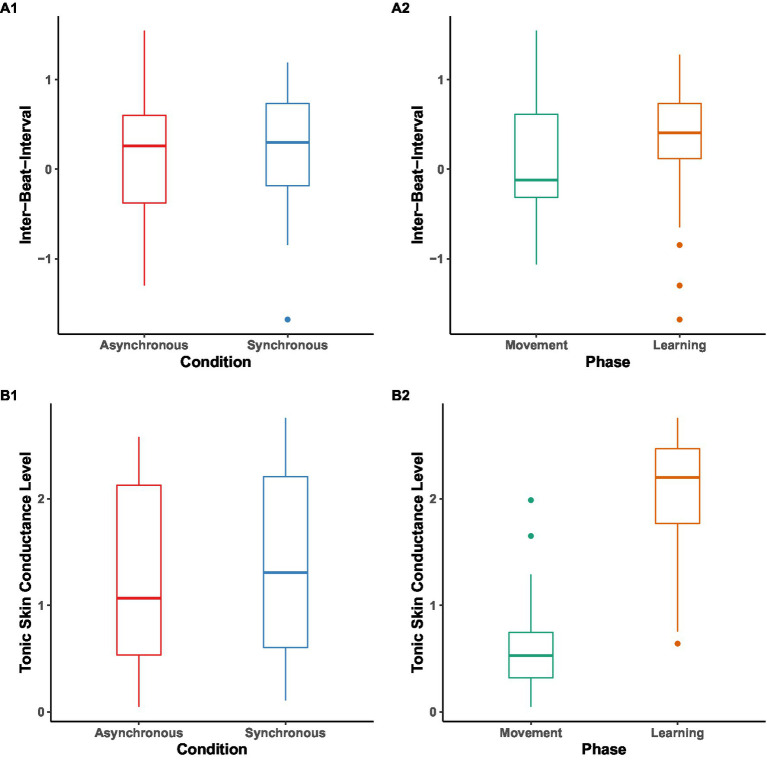
Inter-Beat-Interval (IBI) and Tonic Skin Conductance Level (SCL) baseline-corrected difference scores in z values across phases and conditions. Box plot of IBI (A1, A2) and SCL (B1, B2) across phases and conditions. The midline in the box represents the median values of each condition/phase, outliers are shown as circles, and the error bars represent the 95% CI.

We pre-registered to conduct a 2 (condition: synchronous or asynchronous) × 2 (phase: (a)synchrony movement vs. learning) mixed analysis of variance (ANOVA) on each of the physiological arousal indices. Due to fussiness and/or technical issues, 13 participants had missing or incomplete Inter-Beat-Interval (IBI) data, resulting in a reduced sample size of 27 children (*n*_Async_ = 14, *n*_Sync_ = 13) in this analysis. Similarly, due to fussiness and/or technical issues, 3 participants had missing or incomplete Tonic Skin Conductance Level (SCL) data, resulting in a reduced sample size of 37 children (*n_Async_* = 19, *n_Sync_* = 18) in this analysis. As this relatively small sample size may reduce the power of ANOVA, we instead ran independent sample *t*-tests for arousal indices in two conditions, and pairwise *t*-tests for arousal indices in the movement and learning phases, both to look at the effect of the condition and the effect of the phase (Figure 5). Further, due to the SCL data not being normally distributed, we used the non-parametric alternatives: Mann–Whitney test for SCL between conditions, and Wilcoxon signed-rank test for SCL between phases.

For IBI data, no significant result was found between conditions [*M_Sync_* = 0.19, *SD* = 0.69, *M_Async_* = 0.19, *SD* = 0.73, *t*(52) = 0.002, *p* = 0.998] and phases (*M_movement_* = 0.13, *SD* = 0.69, *M_learning_* = 0.25, *SD* = 0.72, *p* = 0.53).

For SCL data, no significant result was found between conditions (*Mdn_Sync_* = 1.31, *Mdn_Async_* = 1.07, *U = 665, p* = 0.84). However, we found SCL during the learning phase (*Mdn* = 2.20) significantly higher than during movement phase (*Mdn* = 0.53), *V* = 73, *p* < 0.001.

Overall, despite finding higher SCL arousal in learning as compared to the synchronized movement phase (but not higher IBI), we did not find different levels of arousal between conditions, in contrast to our hypothesis 2.

*Hypothesis 3.* Effect of synchrony and arousal on word learning.

We fitted a multiple linear regression as pre-registered to investigate the role of the hypothesized predictors for novel label retention. Due to data loss caused by fussiness and/or technical issues for both IBI and SCL, this regression was conducted with a sample size of 26 participants out of 40 (*n_Async_* = 13, *n_Sync_* = 13). We used the proportion of retained labels as the dependent variable, and age, gender, the proportions of the novel learned words or the familiar words recognized, the group (synchronous or asynchronous), and the IBI and SCL as independent variables. The model yielded no significant results (*p*s > 0.27), suggesting that neither IBI nor SCL, nor any other factors in our model, predicted label retention.

Furthermore, as our main question was whether physiological arousal levels in different condition groups predicted the learning outcome, the pre-registered regression model may not be able to answer this question fully. Therefore, we conducted additional linear regressions separately for IBI and SCL to look at whether the interaction of condition and arousal levels predicted the learning outcome. For each model, we submitted the word retention proportion as a dependent variable, interaction of condition and arousal (IBI or SCL), as well as IBI or SCL as a predictor, along with age and gender. Results revealed a main effect of SCL on the word retention proportion (ß = − 0.17, *p* = 0.050), suggesting increased SCL predicted poorer performance during word retention regardless of condition. Neither main effect of IBI, nor any of the interaction effects, age or gender predictors revealed significance, *p*s > 0.062.

### Exploratory analyses

Given the null results, we conducted two exploratory analyses we did not pre-register to investigate the label retention at the trial level and to look at the individual differences in the physiological arousal indices.

First, instead of the proportion, we used the raw accuracy scores from each of the four trials of the retention test phase (assigning a score of 1 for a correct and 0 for an incorrect response). A generalized linear mixed effects model (GLMM) was fitted with the raw accuracy as a dependent variable, condition (synchronous vs. asynchronous) and the word learning task trial type (novel vs. retained labels selection), as well as their interactions, as fixed effects, and with participant as a random effect. Results revealed a significant main effect of the phase, *X*^2^(2, 40) = 131.71, *p* < 0.001. No other significant results were shown (*p*s > 0.281). This additional test is consistent with the results of the pre-registered analyses.

**Figure 6:**
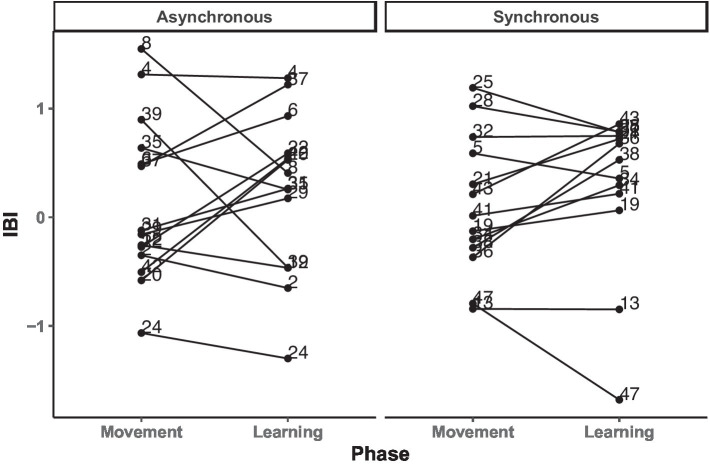
Change in individual participants’ IBI levels across (a)synchronous movement and learning phases, separated by asynchronous and synchronous conditions.

Our second step stemmed from the main analysis showing that the IBI did not significantly differ between conditions or phases, motivating us to further investigate the relationship between heart rate change and novel word retention at the trial level. We computed the Pearson’s correlation between the IBI data and the movement and the learning phases. The results showed a medium positive correlation between IBI across the two phases (*r* = 0.58, *p* = 0.004), suggesting presence of individual differences (see Figure 6).

Next, to clarify whether the individual differences in the IBI changes were influenced by condition, a GLMM was fitted with the IBI difference score as the dependent variable, condition as a fixed effect, and participant as a random effect. Here we calculated the changes in the IBI by using absolute IBI values between the movement and learning phases for each individual. This analysis yielded no significant results, *p*s > 0.22, confirming that the individual differences visually present in changes in the IBI (Figure 6) are not statistically different between conditions.

Finally, to investigate whether label retention was influenced by these individual differences, we split the participants into two groups based on whether the change in their IBI increased or decreased between the movement and the learning phases. A GLMM was fitted with the raw accuracy as the dependent variable, the group assignment based on the direction of the IBI change (increased or decreased) as fixed, and participant as random effect. No significant result was found, *p* = 0.72, indicating that IBI change did not predict children’s performance during test.

## Discussion

Our primary research question was to investigate whether interpersonal behavioral synchrony facilitates young children’s novel word learning. Specifically, our paradigm was designed to test if engaging in a behaviorally synchronous interactive movement with an adult improves toddlers’ novel word retention in a subsequent word learning task with the same adult. We expected that following a synchronous episode, children would successfully retain more novel words than following an asynchronous episode. However, our main results revealed that children’s retention rate did not differ between conditions: although we observed the above-chance retention of novel labels only following the synchronous, but not the asynchronous episode, the comparison between conditions did not reach significance. Second, our goal was to assess if synchronized movement episode affected children’s physiological arousal (namely, the heart rate and skin conductance response), and, thirdly, if physiological indices associated with synchrony would accompany higher rates of successful novel word learning, compared to asynchrony. The results showed that heart rate and skin conductance response did not differ between conditions and did not impact the novel word retention outcome, nor did the proportion of successfully learned words, age, or gender.

To the best of our knowledge, no studies of the direct effect of induced interpersonal behavioral movement synchrony on young children’s learning in direct pedagogical contexts have been reported to date. Despite insufficient support for the null hypothesis, these results contribute to our understanding of interpersonal synchrony experience, albeit in its experimentally imposed form. While its effects on prosocial outcomes are well documented, they do not appear to extend to the specific knowledge acquisition domain. Limited research in adults has explored the effect of synchrony on learning, producing inconclusive findings and exposing substantial methodological variability (Miles et al., 2010; Zheng et al., 2018; Nozawa et al., 2019). Our results, nevertheless, are in line with one directly relevant prior study conducted with adults which reported no effect of interpersonal synchrony on word learning in adult pairs tasked with teaching and learning novel labels from each other (Nozawa et al., 2019). This was despite behavioral and neural alignment in the teacher-learner dyad and establishing positive rapport with each other, which have been posited to enhance the learning outcomes (Hoehl et al., 2021).

Our behavioral results therefore do not supply any evidence for the top-down socio-cognitive mechanism, where prosocial perceptions resulting from the interpersonal synchronous interaction would have transferred to the learning domain. The effect of social bonding or teacher-child closeness on learning outcomes, such as vocabulary (Spilt et al., 2015), literacy and maths (Lowenstein et al., 2015), and academic achievement (Roorda et al., 2017) is abundantly reported in developmental literature. However, our direct experimental test of this hypothesized effect using a synchronized movement as an induction, on a specific learning outcome provided no support that children would have a higher chance of encoding information acquired after a synchronous interaction with a social partner, as opposed to asynchronous.

Several speculative explanations of these null results are possible. First, the result may be due to specific methodological choices, both at the (a)synchrony movement episode and at the learning and retention phases of the word learning task. Synchrony may not have had a direct effect because the learning outcome was unrelated to what was happening during the synchrony episode as such: being rocked to the music and later learning novel labels with unfamiliar objects may have been perceived as unique episodes. Further, the movement episode itself involved caregivers who managed the rocking, rather than children doing it spontaneously or autonomously. Notably, this decision was based on prior successfully applied methodological choices with infants in terms of prosocial outcomes (Cirelli et al., 2014; Tunçgenç et al., 2015; Rabinowitch and Meltzoff, 2017), but it may have prevented the transfer to the label learning domain in this case. It is also possible that the choice of the synchrony episode was crucial, specifically because it was induced and experienced indirectly, with the child being a passive partner. Our focus on such conceptualization of synchrony by design allowed us to look at the synchronized movement *per se*, reasoning that in more naturally occurring, self-induced synchrony, there would be confounding inter- and intra-personal effects, making it hard to isolate the precise effect of entrained, rather than rich and embodied experiences of synchrony. We acknowledge the limits of such conceptualizations yet resorted to this method (also commonly used in synchrony research and across other domains of cognition) to look at the general cognitive mechanism of the effect of synchrony on information retention. Future studies should investigate the variety of paradigms, including those where the child spontaneously engages in a synchronous activity with the teacher.

Similarly, with regards to the learning phase, it may be that our choice of the specific learning outcome was not optimal to detect the hypothesized effect. Here, either synchrony may not affect novel word learning broadly, or it may not affect the type of novel word learning examined in our study, which was based on a fast-mapping process (Horst and Samuelson, 2008) that was self-directed and largely independent from social interaction, rather than being teacher-directed, where learning happens in a top-down manner and may be more prone to social influences. Thus, the current study cannot rule out the effect of synchrony on learning broadly, as there might be an effect under different circumstances, or perhaps only a long-term but not an immediate effect, crystallizing through the social bonding process. In other words, a short 2 minute episode with a stranger may not be a sufficient prime to have a considerable effect on specific learning outcomes.

Finally, the null results might be due to both conditions presenting children with highly interactive embodied social activities, where the adult experimenter displayed equal engagement and positive affect. Such interaction in itself may facilitate the learning experience, with the asynchronous episode therefore not leading to disintegrated learning; hence no pronounced exclusive effect of synchrony was detected in our paradigm. This is corroborated by the very similar rates of novel referent retention reported in the seminal study by Horst and Samuelson (2008), where there was no preceding behavioral induction procedure. All these possibilities are fruitful future directions in this line of research.

Our second research question was whether children’s physiological responses differed during synchronous and asynchronous movement episodes and affected the subsequent learning phase. We found no evidence that the skin conductance response and heart rate indices differed between conditions, nor did they differentially affect word learning. Prior research proposed that physiological processes underlie synchrony, focusing largely on social and affiliative outcomes (Cirelli, 2018; Davis et al., 2018; Kragness and Cirelli, 2021). To match these to the physiological correlates of learning, we chose to look at heart rate as it has been previously shown to relate to attention in infants during object examination (Lansink et al., 2000), preceded changes in looking behavior (de Barbaro et al., 2017), and predicted infant gaze following (Ishikawa and Itakura, 2019). We also chose to look at the skin conductance response as it has been previously implicated in encoding cognitive engagement, effortful allocation of attentional resources, information-seeking, exploration, and curiosity (Berlyne et al., 1963; Spinks et al., 1985; Boucsein, 1993; Critchley, 2002; Nagai et al., 2004; Merrifield and Danckert, 2014; Jang et al., 2015). Thus, we reasoned that heart rate and skin conductance are good candidates to demonstrate the link between synchrony and learning. Our study, however, showed no relationship between interpersonal synchrony and these physiological data.

Our only statistically significant finding was that tonic skin conductance was higher during the learning as compared to the movement phase, irrespective of the (a)synchrony condition. This is consistent with prior research which suggests that an increase in tonic skin conductance level reflects general engagement of attention and increase in cognitive load (Frith and Allen, 1983; O’Connell et al., 2008; Macpherson et al., 2017), as well as a decrease in boredom (Merrifield and Danckert, 2014; Jang et al., 2015). However, this result should also be interpreted with caution as it is expected that tonic skin conductance level rises with time, so this detected main effect of phase might be in fact unrelated to cognitive processes. Similarly, our unexpected, and only marginally statistically significant, result that skin conductance level, regardless of synchrony condition, predicted poorer performance during word retention should be treated with caution.

We thus find no evidence for the hypothesized bottom-up physiological process underlying synchrony. There may be several reasons for the null results. Our choice of physiology data to collect, the tool (Empatica E4 wristband) and the continuous variables averaging arousal across phases that were subjected to analyses may not have been the most sensitive to detect any differences across phases or conditions. This is supported by vast methodological differences and unique considerations pertaining to measures of arousal, such as differential effects in tonic but not phasic measures, lack of relationship between heart rate and skin conductance measures, and between physiological measures and neural or behavioral measures of synchrony (Wass et al., 2015; Mønster et al., 2016; Kragness and Cirelli, 2021; Nguyen et al., 2021). There is also evidence for individual differences in arousal levels and complexities associated with its measurement (e.g., Pijeira-Díaz et al., 2019), contributing to our lack of confidence in these findings.

A number of general limitations are of note in our study. First, although pre-registered based on the power analysis, the sample size was relatively small, which may have also contributed to the insufficient support for either the alternative or null hypotheses on the Bayes Factor analyses. Second, as already mentioned above, the choice of novel word retention as a target outcome and the specific learning task, while well established as valid in the prior literature, may have been too stringent to broadly investigate synchrony and learning. Finally, our physiological measures may not have been sensitive enough to the changes in arousal since the length of the experiment was relatively short and the physiology band only provided continuous but not event-related measurement of the arousal indices, which would have been more fine-grained. In addition, the Empatica E4 tool has not been yet widely validated in developmental research (but see, e.g., Bainbridge et al., 2021) and its treatment of the heart rate data using a proprietary algorithm makes manual correction of movement artifacts and verification of data difficult.

Broadly speaking, with research on synchrony being no exception here, the developmental literature has recently been concerned with issues of validity, reliability, and generalizability of methodological choices (Kominsky et al., 2022; Yarkoni, 2022; Zettersten et al., 2022). Namely, any measure of any psychological construct that cannot be observed or measured directly (e.g., an experience of synchrony, either induced or achieved through personal agency), even with the highest reported reliability, may fail the validity check or be deemed incomplete or narrowly conceived, even if we can measure individual differences with detail and precision. As noted above, we conceptualized and manipulated synchrony for the purposes of the current experimental investigation as an induced, entrained experience, rather than child-led, embodied, and agentic. In other words, synchronized movement in our paradigm was not embedded in the contextual and situated nature of the social interaction and does not take into account individual developmental capacities. We then tested the effect of such synchronized movement episode on a particular learning outcome, in an experimental setting, where inevitably some of these naturalistic contexts could not be preserved. We acknowledge that it is challenging to base the investigations of the broader notions of development and learning on the somewhat reductionist concepts of both synchrony and learning, without accounting for the existing relational factors between the child and their caregiver who formed a dyad in the synchronized movement induction (Feldman, 2006, 2012; Thelen and Smith, 1998; Harrist and Waugh, 2002; Mayo and Gordon, 2020), although at the same time, it allows to isolate the cognitive process. Balancing ecological and measurement validity and accounting for relational and contextual factors is therefore a challenge that future research on the effect of synchrony should aim to tackle.

To shed light on the underlying mechanisms of interpersonal synchrony experience and its effects on learning, future research should make further attempts to evaluate the role of both social learning and physiological mechanisms on attention and cognition. A number of exciting directions are evident in this under-investigated line of work. First, different methodological choices can be explored including a different synchrony prime (e.g., movement, joint action, or music), a different learning task (e.g., one that is not contingent on experimenters’ facilitation, or, instead, directly taught by the adult, and look at both linguistic and non-linguistic outcomes). Second, the effect of synchrony on learning should be tested in a younger age group to better understand the early emerging mechanism. At the same time, more studies with adults could help determine the basis for the existence or lack of this hypothesized effect. Further, physiological arousal should be tested using a variety of reliable measures and tools, including embarking on the analyses of dynamic synchrony of time-synchronized arousal data between the child and the experimenter.

In conclusion, while our work did not produce conclusive results regarding synchrony and word learning in young children, it adds to this growing literature by highlighting the need to investigate both behavioral and physiological arousal indices to better understand the underlying mechanisms of interpersonal synchrony as such, and its possible effect in the knowledge acquisition, rather than prosocial, and domain.

## Data availability statement

The materials and the data that support the findings of this study are openly available on the Open Science Framework (https://osf.io/njgyq/).

## Ethics statement

The studies involving human participants were reviewed and approved by Faculty of Science and Technology Ethics Committee at Lancaster University. Written informed consent to participate in this study was provided by the participants’ legal guardian/next of kin.

## Author contributions

MB, HK, HT, and GW designed the study. HT booked participants and carried out the experiment. MW handled and processed physiological data. HK performed the statistical analyses. MB, HK, and GW wrote the manuscript. All authors contributed to the article and approved the submitted version.

## Funding

This work was supported by the Leverhulme Trust Doctoral Scholarship Programme [DS-2014-14] and the ESRC International Centre for Language and Communicative Development (LuCiD [ES/S007113/1]).

## Conflict of interest

The authors declare that the research was conducted in the absence of any commercial or financial relationships that could be construed as a potential conflict of interest.

## Publisher’s note

All claims expressed in this article are solely those of the authors and do not necessarily represent those of their affiliated organizations, or those of the publisher, the editors and the reviewers. Any product that may be evaluated in this article, or claim that may be made by its manufacturer, is not guaranteed or endorsed by the publisher.

## Footnotes

^1^ https://osf.io/qa5gc

^2^The experimenter’s physiological response was also recorded using the wristband, as pre-registered, however, these data are not being reported here.

^3^ http://imogenheap.com/thehappysong

^4^ https://www.empatica.com/en-gb/manuals/

^5^ http://www.ledalab.de
